# BDNF signaling and survival of striatal neurons

**DOI:** 10.3389/fncel.2014.00254

**Published:** 2014-08-28

**Authors:** Maryna Baydyuk, Baoji Xu

**Affiliations:** ^1^National Institute of Neurological Diseases and Stroke, National Institutes of HealthBethesda, MD, USA; ^2^Department of Neuroscience, The Scripps Research Institute FloridaJupiter, FL, USA

**Keywords:** neurotrophins, BDNF, TrkB, striatum, Huntington’s disease, DRD1a, DRD2, dopaminergic neurons

## Abstract

The striatum, a major component of the basal ganglia, performs multiple functions including control of movement, reward, and addiction. Dysfunction and death of striatal neurons are the main causes for the motor disorders associated with Huntington’s disease (HD). Brain-derived neurotrophic factor (BDNF), a member of the neurotrophin family, is among factors that promote survival and proper function of this neuronal population. Here, we review recent studies showing that BDNF determines the size of the striatum by supporting survival of the immature striatal neurons at their origin, promotes maturation of striatal neurons, and facilitates establishment of striatal connections during brain development. We also examine the role of BDNF in maintaining proper function of the striatum during adulthood, summarize the mechanisms that lead to a deficiency in BDNF signaling and subsequently striatal degeneration in HD, and highlight a potential role of BDNF as a therapeutic target for HD treatment.

## Introduction

The striatum is the largest component of the basal ganglia. It is responsible for movement control and is associated with addictive behaviors. The striatum receives projections from the entire cortical mantle; processes motor, sensory, and associational cortical information; and passes it to the output nuclei of the basal ganglia (Gerfen et al., 1990, 2002; Bolam et al., 2000). Most striatal functions are mediated by the medium-sized spiny neurons (MSNs), which comprise 95% of the striatum with the rest being interneurons (Parent and Hazrati, 1995; Kawaguchi, 1997). MSNs, which use γ-amino butyric acid (GABA) as a transmitter, are born in the ventricular/subventricular zones (VZ/SVZ) of the lateral ganglionic eminence (LGE) and migrate to the striatum during embryogenesis (Hamasaki et al., 2003). They are divided into two equal size populations based on their projection sites and protein expression pattern. The MSNs that form the direct pathway send projections directly to the output nuclei of the basal ganglia, such as the internal segment of the globus pallidus, the substantia nigra pars reticulata, and the ventral pallidum. The other population of the MSNs sits at the origin of the indirect pathway and projects to the output nuclei of the basal ganglia via the external segment of the globus pallidus and the subthalamic nucleus (Bolam et al., 2000). Under normal conditions, activation of the direct pathway leads to initiation of movement. On the other hand, activation of the indirect pathway leads to the opposite physiological effects such as termination of movement or suppression of unwanted movements (Mink and Thach, 1993). In addition to their distinct functions, the MSNs of the two pathways express different sets of neuropeptides and dopamine receptors: neurons of the direct pathway express substance P (SP) and the dopamine receptor D1a (DRD1a), while neurons of the indirect pathway produce enkephalin (Enk) and the dopamine receptor D2 (DRD2; Kawaguchi, 1997). These distinct expression patterns in the MSNs of the two pathways are further confirmed in bacterial artificial chromosome (BAC) transgenic mice expressing fluorescent proteins under the control of the promoter for either DRD1a or DRD2 (Day et al., 2006; Shuen et al., 2008).

Recent studies have shown that striatal neurons are dependent on neurotrophins for their development, survival, and proper function. Increased attention has been given to brain-derived neurotrophic factor (BDNF) and its function in normal and pathological conditions. BDNF is a member of the neurotrophin family, which also includes nerve growth factor (NGF), neurotrophin-3 (NT-3), and neurotrophin-4/5 (NT-4/5). These secreted proteins exert many biological effects by binding and activating specific tropomyosin-related kinase (Trk) receptors. NGF activates TrkA, BDNF and NT4/5 activate TrkB, and NT3 activates TrkC (Reichardt, 2006). Upon binding to BDNF, activated full-length TrkB triggers multiple intracellular signaling cascades through protein-protein interactions (Chao, 2003). The three major pathways, activated by TrkB include: (1) the PLC-γ pathway that leads to production of diacylglycerol and an increase in intracellular calcium, and as a result activation of CAM kinases and PKC; (2) PI-3-kinase pathway that activates AKT, which mediates anti-apoptotic effects; and (3) MAP/ERK pathway that activates regulators of protein translation (Segal, 2003). By activating these diverse signaling cascades in neurons, BDNF can regulate neuronal development and survival, initiation of neurite outgrowth and path-finding (Bhave et al., 1999; Encinas et al., 1999; Yamada et al., 1999, 2001). It can also mediate various synaptic reorganization processes, including formation and maintenance of dendrites and dendritic spines (McAllister et al., 1999; Orefice et al., 2013). Deletion of either the *TrkB* or *Bdnf* gene leads to cell atrophy, dendritic degeneration, and neuronal loss, as shown in the excitatory neurons of the dorsal forebrain (Xu et al., 2000a; Gorski et al., 2003). In addition, BDNF plays an important role in modulating synaptic function and plasticity such as long-term potentiation (LTP), a cellular substrate for learning and memory (McAllister et al., 1999; Poo, 2001).

The vital role of neurotrophins in survival of developing neurons in the peripheral nervous system (PNS) has been well established (Crowley et al., 1994; Smeyne et al., 1994). In the PNS, developing neurons at their final location compete for a limited amount of neurotrophic factors produced by their target tissues; neurons unable to obtain sufficient amounts of trophic factors die via programmed cell death (Zweifel et al., 2005). This mode of survival implies that peripheral target controls the final size of the innervating neuronal population through neurotrophic factors. In contrast, the role of neurotrophins in survival of developing neurons in the central nervous system (CNS) has not been determined until recently. In this review we will discuss our reports, demonstrating that in the developing striatum and LGE, BDNF and NT3 mediate survival of immature MSNs of the indirect and direct pathways, respectively, at the place of their origin before they migrate to their final destination (Baydyuk et al., 2013). Our findings support the idea that a single neurotrophin might be sufficient and necessary to support survival of developing neuronal populations in the CNS. Moreover, we propose a new mode of neurotrophic action in the brain, suggesting that innervating neurons may control the size of their target.

It has been shown that neurotrophins participate in the maintenance of adult neuronal populations in the brain (Xu et al., 2000b; Baquet et al., 2004). A modest increase in postnatal apoptosis was observed in hippocampal and cerebellar granule cells of *TrkB* and *TrkC* knockout mice, however these deletions do not appear to affect the size of these two neuronal populations (Minichiello and Klein, 1996; Alcántara et al., 1997). The redundancy of neurotrophin-mediated signaling pathways in brain regions where more than one Trk receptor is present can provide an explanation for the rather minor effect when one receptor or its ligands are removed. It is also difficult to assess the role of neurotrophins, BDNF in particular, in the postnatal CNS due to lethality of *Bdnf*-null mice in the first two postnatal weeks. Generation of several mouse lines with area-specific *Bdnf* and *TrkB* deletions allowed for the detailed examination of their roles in striatal postnatal growth and maturation. We will review several studies that demonstrate important functions of BDNF-TrkB signaling in promoting somatic growth, dendritic complexity, and spine density in striatal neurons (Baquet et al., 2004; Rauskolb et al., 2010; Li et al., 2012).

Deficiency in BDNF signaling has been linked with an increasing number of conditions that cause brain dysfunction, and the connection between BDNF loss in the striatum and Huntington’s disease (HD) pathology has been extensively investigated. HD is caused by the CAG trinucleotide repeat expansion in the first exon of the gene encoding huntingtin protein (htt; The Huntington’s Disease Collaborative Research Group, 1993). This mutation is translated into a polyglutamine (poly Q) stretch near the amino terminus of htt, which results in a toxic gain of function (Gusella and MacDonald, 2000). Although mutant htt is found throughout the HD brain, the striatum is affected early and more severely during the course of the disease. Striatal atrophy is due to selective degeneration of the MSNs with neuronal loss of 50–60% (Mann et al., 1993; Vonsattel and DiFiglia, 1998). Interestingly, the MSNs of the indirect pathway, responsible for inhibition of involuntary movement, are preferentially affected, causing motor symptoms of HD such as uncontrollable sequence of movements called chorea. The exact mechanism behind selective degeneration of striatal neurons remains to be elucidated, but it has been suggested that reduced trophic support renders striatal neurons more vulnerable to the toxic actions of mutant htt. In support of this view, reduced levels of BDNF protein are detected in the striatum of HD animal models (Spires et al., 2004; Apostol et al., 2008; Gharami et al., 2008) and HD patients (Ferrer et al., 2000). The changes in striatal gene expression profile are similar in HD patients and mice with BDNF deficiency (Strand et al., 2007). Moreover, lack of BDNF-mediated signaling alone is sufficient to cause dendritic abnormalities and neuronal loss in the striatum (Gorski et al., 2003; Baquet et al., 2004), and progression of HD is accelerated in *Bdnf* heterozygous mice (Canals et al., 2004). Importantly, it has been determined that mutant htt decreases striatal BDNF by interfering with BDNF synthesis and transport (Zuccato et al., 2001; Cattaneo, 2003; Gauthier et al., 2004). Furthermore, our recent study shows that the TrkB receptor is selectively expressed in striatal MSNs of the indirect pathway, which may explain why this population of neurons degenerates first in HD patients (Baydyuk et al., 2011). Taken together, these observations raise the possibility that reduced levels of striatal BDNF may significantly contribute to the HD pathogenesis and identify BDNF-TrkB signaling pathway as a potential therapeutic target for HD treatment.

## BDNF and TrkB in the adult and developing striatum: expression patterns

**Figure 1:**
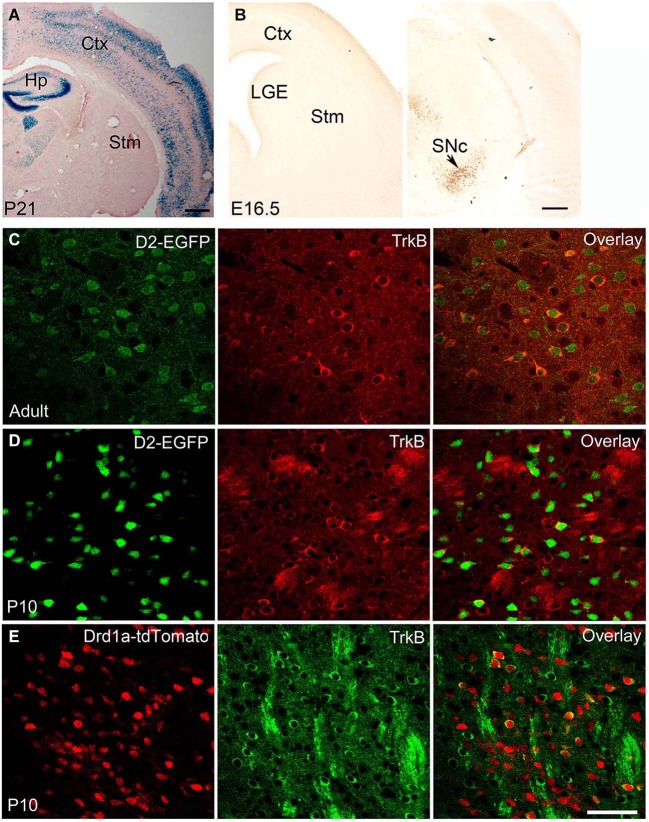
**BDNF and TrkB expression in adult and developing striatum. (A)** BDNF expression in *Bdnf*^LacZ/+^ brain at P21, as revealed by X-gal staining for β-galactosidase (blue). Scale bars, 500 μm. **(B)** BDNF expression in *Bdnf*^LacZ/+^ embryos at E16.5, as revealed by immunohistochemistry to β-galactosidase. The arrow denotes the substantia nigra. Scale bar, 250 μm. **(C)** Co-localization of TrkB with DRD2 in the striatum of adult *TrkB*^LacZ/+^; *D2-EGFP* mice in which β-galactosidase and EGFP serve as indicators for expression of TrkB and DRD2, respectively. Fluorescent immunohistochemistry with antibodies to β-galactosidase and EGFP shows that the majority of TrkB-expressing neurons also express DRD2 in the adult striatum. **(D)** High co-expression of TrkB and DRD2 in the young striatum. Nearly all EGFP^+^ cells expressed β-galactosidase in the striatum of *TrkB*^LacZ/+^; *D2-EGFP* mice at P10. **(E)** Low co-expression of TrkB and DRD1a in the striatum of *TrkB*^LacZ/+^; *Drd1a-tdTomato* mice at P10. Scale bar, 50 μm. Ctx, cerebral cortex; Hp, hippocampus; LGE, lateral ganglionic eminence; SNc, substantia nigra pars compacta; Stm, striatum. This figure is adapted from Baydyuk et al. (2011, 2013).

In the adult brain, BDNF protein is found in many regions, including the cerebral cortex, basal forebrain, striatum, hippocampus, hypothalamus, brainstem, and cerebellum (Conner et al., 1997). In most brain regions, such as cortex, both *Bdnf* mRNA and BDNF protein are present. In contrast, in the striatum *Bdnf* mRNA is virtually absent, whereas BDNF protein levels are high (Figure 1A; Spires et al., 2004; Apostol et al., 2008; Gharami et al., 2008). BDNF found in the adult striatum is synthesized and anterogradely transported from the cell bodies located in the cerebral cortex, substantia nigra pars compacta, amygdala, and thalamus (Altar et al., 1997; Baquet et al., 2004). Since the striatum does not produce BDNF but depends on it for its proper function, abnormalities in anterograde transport and reduced gene expression from brain regions supplying BDNF to the striatum might cause neuronal dysfunction and striatal atrophy.

The expression levels of BDNF fluctuate during development. In rodents, BDNF is expressed by a number of cells in the hippocampus on embryonic day 15.5 (E15.5), and by E17 it is found in the piriform cortex, hippocampus, thalamus, hypothalamus and amygdala, but in only a few cells in the cortex and none in the striatum (Baydyuk et al., 2013). Cortical expression appears at postnatal day 4 (P4) and increases with age, reaching the adult levels by P21 (Baquet et al., 2004). The corticostriatal connections are also mainly formed during the first postnatal week (Nisenbaum et al., 1998; Christensen et al., 1999). Thus, it is unlikely that the cerebral cortex is the main source of BDNF for immature neurons in the LGE, in contrast to the adult striatum. Since BDNF is not expressed in the LGE and the nearby striatum (Figure 1B), but BDNF protein is already present in this region at E16.5 (Baydyuk et al., 2013), it likely comes from other brain regions that express BDNF during embryogenesis and form connections with the LGE and the developing striatum.

It is equally important to note that since immature neurons in the LGE have to migrate to the striatum before they send axons to striatal targets, it is impossible for them to obtain BDNF from their eventual targets through retrograde transport. Therefore, the only source of BDNF in the LGE is from axons of neurons that project to this region. In our recent study, we show that BDNF is expressed in the substantia nigra at P0 and E16.5 specifically in neurons positive for tyrosine hydroxylase (TH; Baydyuk et al., 2013; Figure 1B). It has also been shown that nigrostriatal projections are formed by E16.5 (Voorn et al., 1988; Hu et al., 2004). Furthermore, TH-positive axons are found in close proximity to dividing LGE progenitor cells as early as E13 (Ohtani et al., 2003). These expression and projection findings indicate that BDNF from mesencephalic dopaminergic neurons is the main source of BDNF in the developing striatum and the LGE.

The TrkB receptor is also widely expressed in the developing and adult brain. The TrkB mRNA is first detected in the neuroepithelium and in the neural crest at E9.5 (Klein et al., 1990). During development the TrkB is present at high levels throughout the brain with regional and cell type specific fluctuations. In adulthood, TrkB expression is also confined to specific regions or neuronal subtypes in a complex pattern. Both TrkB mRNA and protein are present in the developing and adult striatum (Yan et al., 1997). Interestingly, TrkB is not equally distributed in all striatal neurons. Instead, it is preferentially expressed by DRD2-expressing MSNs of the indirect pathway (Figures 1C,D), with 98% of DRD2 MSNs expressing TrkB at P10 and 43% in adult animal (Baydyuk et al., 2011). The observed preferential TrkB expression may provide insights into the selectivity of degeneration associated with HD, where a greater decrease in the number of DRD2 MSNs is observed (Reiner et al., 1988). These neurons act to terminate movement associated with the basal ganglia or suppress unwanted sequences of movements (Bolam et al., 2000). Hence, the loss of the indirect pathway neurons leads to disinhibition of the thalamus and increased facilitation of the motor cortex, producing hyperkinesias in HD patients (Calabresi et al., 2000). Direct pathway neurons that express DRD1a are less affected, and striatal interneurons are mostly spared in patients with HD (Ferrante et al., 1987a,b). In agreement with these facts, only 18% of DRD1a MSNs express TrkB at P10 (Figure 1E, Baydyuk et al., 2011). These findings indicate that decreased BDNF-TrkB signaling may preferentially affect indirect pathway MSNs, which express most of the TrkB in the striatum, therefore explaining the selective degeneration of this population and motor phenotype seen in HD.

## Role of BDNF and NT3 in survival of developing striatal neurons at their origin

Striatal projection MSNs originate in the VZ/SVZ of the LGE between E12 and P2 with a peak around E15, and subsequently migrate along guiding radial glia into the striatum (Marchand and Lajoie, 1986; Olsson et al., 1995, 1998). Several factors have been identified as regulators of striatal neurogenesis and fate determination. For instance, dopamine coming from the substantia nigra pars compacta and arriving in the LGE via nigrostriatal pathway as early as E13 (Voorn et al., 1988; Hu et al., 2004) has been shown to modulate striatal neurogenesis by influencing the cell cycle of progenitor cells in the LGE (Ohtani et al., 2003). Marked progress has also been made in understanding cell fate determination of striatal neurons. Several transcription factors have been identified to play a role in differentiation of neuronal precursors into striatal neurons, including Gsh-2, Mash1, and Dlx family members (Dlx1, Dlx2, Dlx5, and Dlx6) (Anderson et al., 1997; Casarosa et al., 1999; Corbin et al., 2000; Stenman et al., 2003). However, until recently it was unclear whether neurotrophins play any role in neuronal survival, target innervation, dendritic arborization, and synaptogenesis of developing striatal neurons.

In our recent studies, we demonstrate that neurotrophins are major players in determining striatal size by promoting survival of newborn striatal neurons. Selective deletion of TrkB in the striatal progenitors, using the *Dlx5/6-Cre* transgene (*TrkB^Dlx^*), leads to a 50% loss of the MSNs, among which DRD2 population is most affected with up to 80% loss, while DRD1a MSNs suffer only 22% loss (Baydyuk et al., 2011). As we discussed above this selective loss of the MSNs in the indirect pathway is likely due to the preferential expression of the TrkB receptor by this population (Figures 1C–E). The magnitude of the observed neuronal loss in *TrkB^Dlx^* mutants is similar at P0 and P21, ruling out the possibility of cell death during postnatal development. We also show that TrkB signaling does not affect striatal neurogenesis, in contrast to the cerebral cortex, where its role in promoting proliferation and differentiation of neuronal precursors is well established (Bartkowska et al., 2007). Instead, we observe numerous apoptotic cells in the striatal VZ/SVZ of *TrkB^Dlx^* mice, indicating that TrkB signaling is essential for survival of developing striatal neurons at their origin (Baydyuk et al., 2011).

In agreement with our previous report, global deletion of BDNF produces similar phenotype, showing selective loss of DRD2 MSNs and the majority of cell death occurring within the LGE during embryogenesis (Baydyuk et al., 2013). These results confirm a crucial role of BDNF-TrkB signaling in survival of developing MSNs of the indirect pathway and indicate that developing MSNs need trophic support before they migrate to the striatum and send out axons.

**Figure 2:**
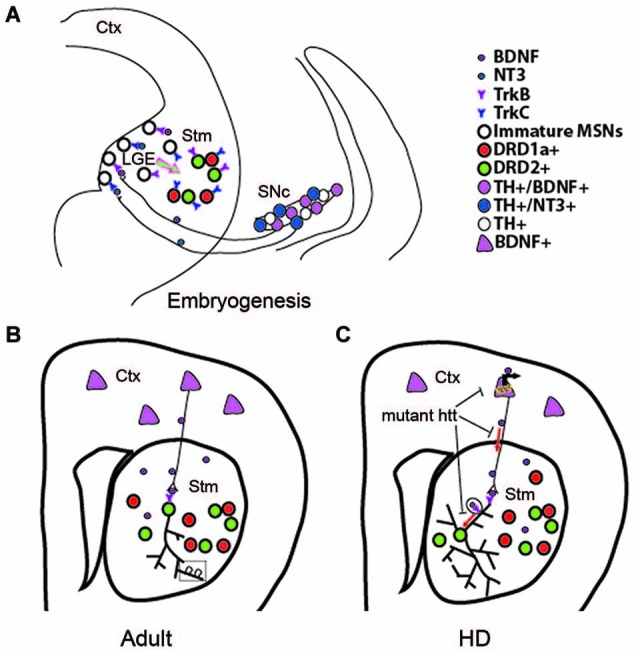
**Role of BDNF in developing, adult, and HD striatum. (A)** A proposed model showing that BDNF and NT3 anterogradely transported from mesencephalic dopaminergic neurons regulate survival of immature neurons in the indirect and direct pathways, respectively. Ctx, cerebral cortex; Stm, striatum; SN, substantia nigra. **(B)** Cortical BDNF in the adult striatum mediates dendritic complexity and spine number and morphology. **(C)** Mutant htt reduces BDNF-TrkB signaling by inhibiting BDNF gene transcription, axonal transport of vesicles containing BDNF, retrograde dendritic transport of TrkB-positive endosomes to the cell body. **Panel A** is adapted from Baydyuk et al. (2013).

MSNs of the direct pathway are not affected by deletion of TrkB or BDNF. Interestingly, signaling via another neurotrophin, NT3, plays an important role in survival of this neuronal population. Both NT3 and TrkC proteins are present in the developing striatum, where 86% of DRD1a-expressing neurons are positive for TrkC. Deletion of either TrkC or NT3 leads to ~30% loss of total number of striatal neurons, with 35% reduction in the number of the MSNs of the direct pathway, whereas MSNs of the indirect pathway are not significantly affected (Baydyuk et al., 2013). Thus, the DRD1a-expressing MSNs of the direct pathway are dependent on the NT3-TrkC signaling for their survival during development. Similar to BDNF, NT3 is not expressed in the LGE or the striatum. Both BDNF and NT3 are produced in the midbrain dopaminergic neurons and anterogradely transported via nigrostriatal pathway to the LGE and striatum (Figure 2A). Deletion of either BDNF or NT3 specifically in TH-positive dopaminergic neurons leads to selective loss of DRD2 or DRD1a MSNs, respectively (Baydyuk et al., 2013).

Together, these findings establish a novel mode of neurotrophin actions in the CNS. It is distinct from the one that has been extensively studied in the PNS, where neurons depend on neurotrophins after they reach their final position and form connections with their targets, competing for a limiting amount of target-derived neurotrophins for their survival. These neurotrophin molecules are internalized at axonal terminals and retrogradely transported to the cell bodies to activate pro-survival signaling cascades (Zweifel et al., 2005). In this way, target tissue determines the final size of the innervating neuronal population. In some cases, PNS neurons also depend on neurotrophins during neurogenesis before target innervation is complete (Fariñas et al., 1998; Coppola et al., 2001). Notably, neurotrophins required for proper formation of these neurons are highly expressed in the close vicinity of developing neurons and growing axons (Fariñas et al., 1996, 2001). In contrast, in the developing striatum survival of immature MSNs at the place of their origin depends on neurotrophins, anterogradely transported from the midbrain dopaminergic neurons (Figure 2A). Thus, we propose that innervating neurons can regulate the size of their targeting neuronal population in the CNS by regulating their survival through neurotrophin release at axonal terminals during embryogenesis and early postnatal development. As a result, nigrostriatal dopaminergic neurons, a relatively small population, can regulate the size of their large target, the striatum, by controlling the survival of immature striatal neurons at their origin.

## Role of BDNF in striatal postnatal growth, maturation, and maintenance

To assess the role of BDNF-TrkB signaling in the postnatal development and maintenance of the striatum, several conditional BDNF knockouts have been generated. Since postnatal striatal BDNF arrives mainly from the cortex, where its expression starts during first postnatal week, cortical ablation of BDNF provides a tool to study a role of BDNF-TrkB signaling in the postnatal striatal growth. Using this approach, Baquet et al. (2004) demonstrate that the striatum of postnatal forebrain-specific *Emx-Bdnf* knockout mice is reduced in volume compared with controls, and the MSNs have shrunken cell somas, thinner dendrites, and fewer dendritic spines at P35. Although significant striatal neuronal loss is not detected at P35 or P120, 35% of striatal neurons are lost in *Emx-Bdnf* knockout mice aged beyond 1 year. Thus, cortical BDNF has a relatively minor role in determining the number of the MSNs early in life but is required for a long-term survival and is necessary for normal dendritic morphogenesis when BDNF expression rises in the cortex (Baquet et al., 2004).

Maturity of striatal neurons is determined by several factors, including expression levels of neuronal markers, complexity of dendritic arbor, and dendritic spine morphology. Studies using global BDNF deletion in the CNS show that BDNF ablation leads to reduction in striatal volume, dendritic complexity, and spine density, indicating impaired striatal maturation in these mutants (Rauskolb et al., 2010; Li et al., 2012). At the cellular level, DARPP32 (dopamine- and cAMP-regulated phosphoprotein, 32 kDa) is a well-established marker of differentiated striatal MSNs, and a key mediator of dopamine signaling (Svenningsson et al., 2004). Mutant mice with BDNF deletion show a marked reduction in DARPP32 expression levels and distribution (Baydyuk et al., 2011; Li et al., 2012). Decrease in dendritic spine number and size in BDNF mutant mice indicate deficits in formation of corticostriatal synaptic connections, as dendritic spines are the main targets of excitatory synapses formed by cortical afferents (Baquet et al., 2004; Rauskolb et al., 2010; Li et al., 2012). These observations indicate that BDNF is an important factor for establishing and maintaining corticostriatal synapses and can influence their strength (Figure 2B).

Morphological, cellular, and functional changes observed in the striatum of *Bdnf* and *TrkB* mutants are often accompanied by behavioral abnormalities. For instance, mutant mice with either forebrain-specific deletion of BDNF or TrkB deletion in striatal progenitors, display hindlimb and forelimb clasping phenotype, which has also been observed in transgenic lines with motor dysfunction or degeneration, including HD mouse models (Baquet et al., 2004; Baydyuk et al., 2011). Deletion of the *Bdnf* gene in the dopaminergic neurons causes cell deaths of the MSNs of the indirect pathway and leads to poor performance on the rotarod, reinforcing the importance of this pathway in motor coordination (Baydyuk et al., 2013). In agreement with this finding, ablation of TrkB selectively from these MSNs results in augmented spontaneous and drug-induced hyper-locomotion, further indicating the importance of BDNF-TrkB signaling in the MSNs of the indirect pathway, which control inhibition of locomotor behavior (Besusso et al., 2013). Importantly, a disrupted expression of *Bdnf* in the HD mouse models advances the onset of motor dysfunctions and produces more severe uncoordinated movements. In all the studies discussed above, the behavioral pathology correlates with specific degeneration of the MSNs of the indirect pathway (Canals et al., 2004).

## Deficiency in BDNF-TrkB signaling and HD: molecular mechanisms and therapeutic implications

### Mutant htt alters BDNF gene expression

The pathogenic mechanisms of HD are not fully understood but are thought to involve the gain of toxic function and/or the loss of normal activities of htt protein (Borrell-Pagès et al., 2006). The combination of both mechanisms can lead to a decrease in striatal BDNF by interfering with BDNF synthesis and transport (Zuccato et al., 2001; Cattaneo, 2003; Gauthier et al., 2004). Wild-type htt is known to regulate transcription of multiple genes, including *Bdnf* (Zuccato et al., 2001). In rodents and humans, the *Bdnf* gene is transcribed from at least eight discrete promoters, producing different *Bdnf* mRNA species that encode the same protein (Aid et al., 2007). The transcripts are generated in different tissues in a stimulus- and development-specific manner and may have differential subcellular localizations and targets (Metsis et al., 1993; Timmusk et al., 1993; Pattabiraman et al., 2005). Zuccato et al. (2001) show that wild-type htt enhances *Bdnf* transcription from promoter II, whereas mutant htt suppresses *Bdnf* transcription from promoter II as well as two other *Bdnf* promoters in cultured cells and the cerebral cortex of YAC72 transgenic mice expressing mutant htt with an expanded tract of 72 glutamines. Wild-type htt promotes transcription of promoter II by sequestering the repressor element-1 transcription factor/neuron restrictive silencer factor (REST/NRSF) in the cytoplasm, thereby freeing the nucleus of the inhibitory complex and allowing transcription to occur (Zuccato et al., 2003). In contrast, mutant htt is unable to retain REST/NRSF complex in the cytoplasm, leading to aberrant accumulation of REST/NRSF in the nucleus and inhibition of *Bdnf* gene transcription.

In agreement with these findings, levels of *Bdnf* mRNA are reduced in the cerebral cortices of HD patients (Zuccato et al., 2008). It also has been shown that lower levels of BDNF are associated with higher numbers of CAG repeats in mutant *htt* alleles and correlate with the severity of the disease (Ciammola et al., 2007). However, this autopsy data should be interpreted with caution. As mutant htt alters electrophysiological properties of cortical neurons (Cummings et al., 2009) and neuronal activity regulates *Bdnf* gene expression (Aid et al., 2007), we should not exclude the possibility that the observed reduction in cortical *Bdnf* mRNA levels may be secondary to neurodegeneration.

Although most findings are in agreement with the notion that both mechanisms, suppressed *Bdnf* gene expression and deficient BDNF transport, might concomitantly contribute to reduced levels of BDNF in the striatum of HD patients and mouse models, several discrepancies still exist between reports on transcriptional regulation of BDNF by htt. A reduction in *Bdnf* transcription would predict reduced levels of BDNF protein in cerebral cortices of both HD patients and mouse models. This prediction has been confirmed in one study (Zuccato et al., 2008) but not in another study (Gauthier et al., 2004), which uses post-mortem tissues from multiple control subjects and HD patients. Furthermore, *in situ* hybridization revealed normal levels of cortical *Bdnf* mRNA in aging YAC128 mice that express the whole human *htt* gene with 128 CAG repeats (Xie et al., 2010). Consistent with this observation, levels of cortical BDNF in YAC128 mice and R6/1 mice, another HD model, were found to be similar to those in WT mice (Gharami et al., 2008; Xie et al., 2010). Despite the variations in determining cortical *Bdnf* mRNA levels, a significant reduction in striatal BDNF has been consistently shown in both HD patients and animal models, thus providing a strong evidence for BDNF as a crucial factor in the pathogenesis of HD.

### Mutant htt inhibits BDNF and TrkB transport

In addition to controlling *Bdnf* mRNA production in the cortex, wild-type htt also regulates BDNF transport along the corticostriatal axes (Figure 2C), the main supply line of BDNF in the adult striatum (Gauthier et al., 2004). Although present in the nucleus, htt is predominantly found in the cytoplasm, where it interacts with huntingtin-associated protein-1 (HAP1), a protein involved in axonal transport via association with p150^glued^ subunit of dynactin, which is an essential part of the microtubule-based motor complex (Block-Galarza et al., 1997; Engelender et al., 1997; Li et al., 1998). Gauthier et al. (2004) show that under normal conditions wild-type htt promotes neuronal survival by facilitating the transport of BDNF-containing vesicles along microtubules. Consistent with a loss-of-function hypothesis, reduction in wild-type htt levels leads to attenuated BDNF trafficking. On the other hand, mutant htt binds tightly to p150^glued^ via HAP1 and prevents efficient movement of BDNF-containing vesicles. Disruption of BDNF transport leads to decreased neurotrophic support and neurotoxicity, which can be rescued by wild-type htt (Gauthier et al., 2004).

Considering the important role of htt in axonal trafficking of BDNF-containing vesicles, it has been recently proposed that htt can also play a role in retrograde transport of the TrkB in striatal dendrites (Figure 2C). It has been shown that upon BDNF binding, TrkB-positive endosomes undergo dynein-dependent retrograde transport along microtubules to the cell body where TrkB induces survival signals (Watson et al., 1999; Heerssen et al., 2004; Ha et al., 2008), a mechanism studied primarily in axons. Recent technical advances that allow to selectively isolate dendrites from the cell bodies using microfluidic devices (Cohen et al., 2011), facilitated the investigation of the TrkB vesicular retrograde transport in control and HD striatal neurons. A study by Liot et al. shows that upon BDNF stimulation, TrkB binds to htt and dynein in dendrites of cultured striatal neurons, and wild-type htt promotes TrkB transport via dynein-dependent mechanism. In striatal neurons from HD mouse model, mutant htt alters binding of TrkB-containing vesicles to microtubules and reduces TrkB retrograde transport in dendrites, leading to reduced BDNF/TrkB signaling (Liot et al., 2013). These findings demonstrate that in addition to affecting BDNF axonal corticostriatal transport, mutant htt can also impair retrograde trafficking of the TrkB-positive endosomes from striatal dendrites to the cell bodies. This transport alteration may further impair BDNF-TrkB signaling, which plays a crucial role in the survival and maintenance of striatal neurons (Baydyuk et al., 2011).

### BDNF rescues HD phenotype—therapeutic implications

The multiple lines of evidence discussed above indicate that reduction in striatal BDNF signaling plays a pivotal role in the pathogenesis of HD. As a result, efforts have been made to examine whether increasing BDNF expression may be a viable strategy for treating HD. Indeed, increasing striatal BDNF levels via pharmacological or behavioral stimulation that induces *Bdnf* gene expression (Duan et al., 2003; Spires et al., 2004; Peng et al., 2008; Simmons et al., 2009) or by viral delivery (Cho et al., 2007), improves disease phenotypes in several HD mouse models. Early symptoms in HD patients are manifested by cognitive and memory deficits that precede the characteristic motor dysfunction (Lawrence et al., 1998; Ho et al., 2003). Similarly, in HD mouse models, impaired learning and memory, measured as hippocampal LTP, occur prior to motor deficits and neuronal loss (Murphy et al., 2000; Mazarakis et al., 2005; Van Raamsdonk et al., 2005). Since BDNF has been shown to modulate LTP, the reduced levels of BDNF in HD patients and mice can disrupt synaptic changes important for learning and memory formation. Applying low concentrations of BDNF to hippocampal slices prepared from HD mice fully restores LTP (Lynch et al., 2007). Furthermore, up-regulation of endogenous BDNF levels with an ampakine, a positive modulator of AMPA-type glutamate receptors, rescues synaptic plasticity and reduces learning deficits in HD mice (Simmons et al., 2009).

In addition to promoting survival and inducing synaptic plasticity, BDNF also regulates adult neurogenesis (Scharfman et al., 2005; Henry et al., 2007; Bath et al., 2011), which appears to be altered in HD mouse models and human postmortem brains (Curtis et al., 2003; Gil et al., 2005; Phillips et al., 2005). Adenoviral delivery of BDNF to the striatum of R6/2 HD mice induces striatal neurogenesis (Cho et al., 2007). Majority of the newly born neurons differentiate to MSNs and become functional, delaying motor impairment and prolonging survival in R6/2 mice. Similar improvements have been seen in the same HD mouse model after administration of the antidepressant sertraline (Peng et al., 2008). By increasing BDNF levels and stimulating neurogenesis, sertraline treatment results in improved motor performance, reduced striatal atrophy, and prolonged survival.

To directly evaluate the effect of increased cortical BDNF supply to the striatum on the progression of HD, our group and others have examined the consequences of overexpression of BDNF in the cortex. In our studies, we employ a *Bdnf* transgene under the control of the promoter for Ca^2+^/calmodulin-dependent protein kinase II alpha (Gharami et al., 2008; Xie et al., 2010). This transgene starts to express BDNF in the cerebral cortex in the first postnatal week and reaches plateau in the third postnatal week, as does the endogenous *Bdnf* gene (Huang et al., 1999). It also expresses at low levels in the striatum where the endogenous *Bdnf* gene is mostly inactive (Gharami et al., 2008; Xie et al., 2010). We show that the *Bdnf* transgene is able to greatly increase BDNF levels in the striata of two HD mouse lines, R6/1 and YAC128, indicating that BDNF overexpressed in the cortex is efficiently transported to the striatum, despite the presence of mutant htt. Importantly, BDNF overexpression reverses brain atrophy, normalizes the expression of several important genes in the striatum, and ameliorates deficits in motor coordination in these two HD mouse models (Gharami et al., 2008; Xie et al., 2010). In addition, overexpression of BDNF in YAC128 mice prevents loss of striatal neurons, normalizes spine abnormalities of MSNs, and significantly improves procedural learning (Xie et al., 2010). In summary, these studies suggest that increasing striatal BDNF levels may have therapeutic potential for HD.

## Concluding remarks

We have described the important roles of neurotrophic signaling in the developing, adult, and diseased striatum. We have examined a novel model of neurotrophic signaling in the developing striatum, where neurotrophins, anterogradely transported from the midbrain dopaminergic neurons, provide a vital support for immature neurons at their origin. However, it remains unclear whether the mechanisms of action for neurotrophins derived from the target tissue as seen in the PNS or transported from the innervating neurons as seen in the striatum, differ from one another. A comprehensive examination of signaling cascades affected by abolishing BDNF-TrkB signaling in the developing and adult striatum could provide some insights into this question. Another major point raised in this review is that BDNF-mediated neuronal survival is occurring early in development, at the time when neurons are being generated, and before mature connections are formed. During postnatal development, BDNF arriving via anterograde axonal transport promotes striatal maturation and influences synaptic connectivity. Thus, BDNF signaling plays various roles at different stages of neuronal development.

Numerous studies presented in this review undoubtedly link BDNF loss in the striatum and HD pathogenesis. Currently, drugs used to treat HD act on the symptoms and do not slow or stop the disease progression. Attempting to restore striatal BDNF levels or activate downstream signaling pathways may have therapeutic potential in treating HD patients. Indeed, multiple lines of evidence, discussed above, suggest that restoring cortical expression, axonal transport, and release of BDNF in the striatum promotes neuronal survival and improves behavioral phenotypes in HD animal models. These findings indicate that increasing BDNF signaling may also overcome functional deficits observed in HD patients.

## Conflict of interest statement

The Reviewer Dr. Tressarollo declares that, despite having collaborated with the authors, the review process was handled objectively. The authors declare that the research was conducted in the absence of any commercial or financial relationships that could be construed as a potential conflict of interest.
